# On the Early and Affordable Diagnosis of Joint Pathologies Using Acoustic Emissions, Deep Learning Decompositions and Prediction Machines

**DOI:** 10.3390/s23094449

**Published:** 2023-05-02

**Authors:** Ejay Nsugbe, Khadijat Olorunlambe, Karl Dearn

**Affiliations:** 1 Nsugbe Research Labs, Swindon SN1 3LG, UK; 2 Mechanical Innovation and Tribology Group, Department of Mechanical Engineering, School of Engineering, University of Birmingham, Birmingham B15 2TT, UK

**Keywords:** AE, signal processing, orthopaedics, joint wear, deep learning, LSDL, epidemiology

## Abstract

The condition of a joint in a human being is prone to wear and several pathologies, particularly in the elderly and athletes. Current means towards assessing the overall condition of a joint to assess for a pathology involve using tools such as X-ray and magnetic resonance imaging, to name a couple. These expensive methods are of limited availability in resource-constrained environments and pose the risk of radiation exposure to the patient. The prospect of acoustic emissions (AEs) presents a modality that can monitor the joints’ conditions passively by recording the high-frequency stress waves emitted during their motion. One of the main challenges associated with this sensing method is decoding and linking acquired AE signals to their source event. In this paper, we investigate AEs’ use to identify five kinds of joint-wear pathologies using a contrast of expert-based handcrafted features and unsupervised feature learning via deep wavelet decomposition (DWS) alongside 12 machine learning models. The results showed an average classification accuracy of 90 ± 7.16% and 97 ± 3.77% for the handcrafted and DWS-based features, implying good prediction accuracies across the various devised approaches. Subsequent work will involve the potential application of regressions towards estimating the associated stage and extent of a wear condition where present, which can form part of an online system for the condition monitoring of joints in human beings.

## 1. Introduction

### 1.1. Joint Wear and Its Epidemiology

**Figure 1:**
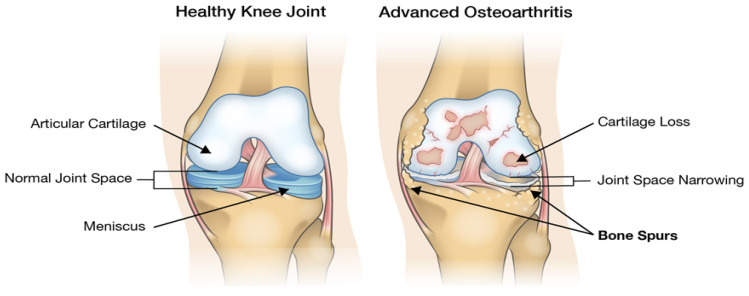
Visual comparison of a healthy and worn knee [4].

It is estimated that there are around 360 joints in the human body, the majority of which are subject to wear and degradation as human beings age, depending on their professions and lifestyle, where problems such as chronic pain and osteoarthritis occur as a result [1]. Further, it has been noted that the world population is ageing and is projected to rise further by the year 2050. From a joint and structural perspective, the implication is that joint-wear problems can be even more prevalent due to the ageing population [2]. For example, one of the noticeable results of joint wear is osteoarthritis, which manifests itself with erosive behaviour of the joint cartilage due to repeated trauma or considerable injury [3]. During osteoarthritis, metabolic changes occur within the joint due to the continued wear, which forms nano/micro-scale fractures that regenerate to form osteophytes, resulting in the knee producing additional fluid, which causes further pain, discomfort and swelling [1,3]. The accumulation of the factors above eventually manifests biomechanically as structural instability, misalignments and deformity. A visual comparison of a healthy knee with a worn knee can be seen in Figure 1, from which it can be noted that there is a substantial amount of cartilage loss, joint-space narrowing and the production of bone spurs.

In addition to ageing individuals, athletes involved in high-impact sports, such as basketball and dancers, have been seen to carry a degree of risk of developing joint-based diseases related to overall wear and osteoarthritis later in life [5]. The early detection of these joint-wear pathologies can allow for proactive care strategies to be offered to patients, thus negating the need for orthopaedic-based surgeries on the joints [5].

### 1.2. Means of Diagnosing Joint Pathologies, Their Shortcomings and AE

Traditionally, the means for diagnosing and identifying joint pathologies is using X-ray imaging, although this method has been critiqued for mostly projecting a cross-section of the bones in question [6]. Computed tomography (CT) is an improvement on traditional X-ray, as it offers a three-dimensional projection of joints and bones, but still exposes the patient to harmful rays during the imaging process and does not give reliable insights into soft tissues, ligaments or muscles [7]. The emergence of dynamic measurements such as magnetic resonance imaging (MRI) has offered a means towards producing an image-based diagnosis of both joint and soft-tissue pathologies, but has shortcomings based on portability, power consumption and overall costs. Thus, their acquisition and use are specialised [8]. Although ultrasound imaging offers a cheaper alternative to MRI—whilst offering an image-based perspective for both joints and soft tissues—its use is limited in diagnosing joint wear and pathologies due to being prone to noise and unable to detect micro- and hairline fractures [9].

Seminal work has been done by researchers who have attempted to track the sound waves—known as biomechanical acoustic emission (AE) waves—that are produced when various joints are flexed, resulting from tissue changes during deformations and movements, and provided signs to suggest that AE can be applied as a means towards assessing the level of degeneration in a joint [10,11,12,13]. The conceptual physics is based on the expectation that the AE events generated by a healthy joint would be less than that of one with a considerable degree of wear within it and can serve as a non-invasive means towards condition monitoring of joint wear [10,11,12,13].

Contrasting the AE sensing approach to clinical joint diagnosis techniques, it is a passive technique that poses minimal risk of infection and radiation and is affordable, as well as being a non-invasive means towards the acquisition of rich signal information that can be utilised towards the diagnosis and assessment of joint health [14]. AE has predominantly seen traditional use in condition monitoring and maintenance of machinery components, in addition to process monitoring, but recent scientific advancements have seen applications of the technique in areas such as biomechanics and orthopaedics, as mentioned [10,11,12,13,14].

Tribological research work by Olorunlambe et al. [10,11,15] identified the notion that there mainly exists a constrained array of signal processing techniques applied towards ortho-tribological case studies, which they built upon by developing machine learning models that could differentiate between adhesive and abrasive wear conditions. The work by Olorunlambe et al. [10,11,15] showcased how machine learning-based methods could distinguish between these two wear conditions from benchtop-based wear conditions in their running in early and steady-state (continuous) stages of wear. The research presented in this paper aims to build on this by designing pattern recognition-based predictive models that can identify and differentiate between the latent and early stages of joint-wear pathologies, a broad class of common joint-wear conditions that includes abrasive, adhesive, burnishing, burnishing-to-scoring transition, and scoring. These results are the first time AE has been applied to investigate and recognise such a broad class of wear conditions. We aim to investigate this problem by applying and comparing two signal processing methods, including a concatenation of a broad list of manually extracted (handcrafted) and an unsupervised feature extraction method from deep wavelet scattering (DWS). DWS represents a fusion between wavelet transform and deep learning, which can extract multiscale features in an unsupervised manner without requiring expert knowledge for the feature extraction stage [16]. As per published literature, this combination also presents the first time that an advanced feature extraction method is contrasted with a multiscale unsupervised feature extraction approach for modelling and characterising a broad range of joint pathologies.

Thus, the explicit novelty and contributions offered as part of this manuscript are as follows:

- the seminal use of AE towards the early diagnosis and differentiation between five different kinds of joint pathologies
- a contrast between an expanded handcrafted feature extraction scheme and an unsupervised multiscale feature extraction method for the characterisation of a candidate AE signal
- an exploratory observation of the optimal machine learning model for this sort of data and pattern recognition exercise through the training and validation of 12 different candidate machine learning models

From this, it is hypothesised that an optimal underlying technical pathway that can be used towards an AE-driven joint pathology monitoring system can be identified and fleshed out, allowing a real-time, affordable and non-invasive method towards monitoring joint wear in orthopaedic medicine and allowing for proactive care interventions.

## 2. AE Sensing

**Figure 2:**
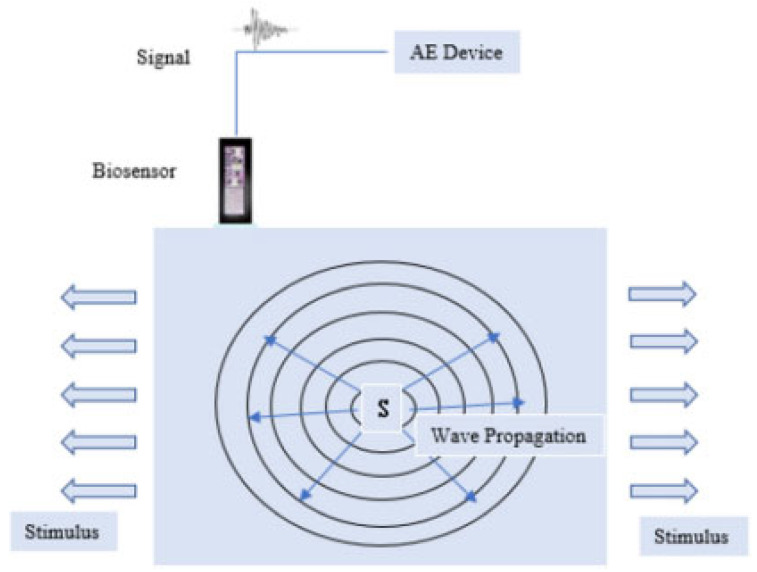
Visual illustration of the propagation of an AE signal [15].

**Figure 3:**
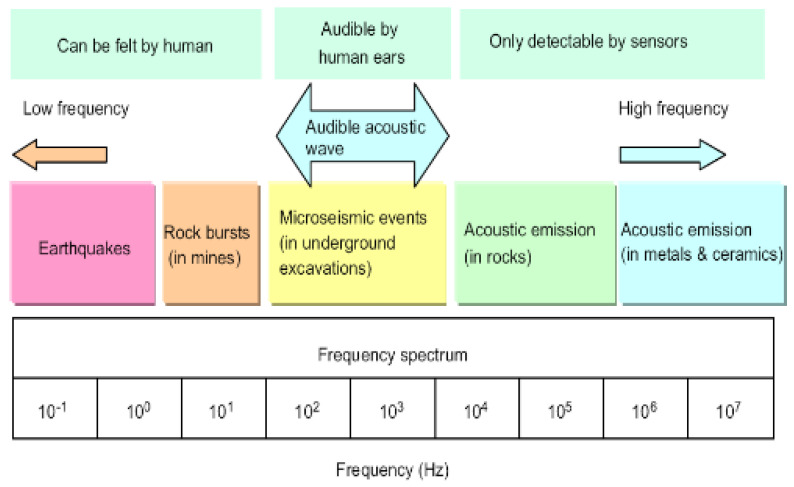
The acoustic spectrum [17].

The concept behind AE sensing is based on the use of a passive monitoring technique capable of recording source events that emanate from events of various kinds during energy release [15]. The said energy release is known to travel in the form of stress waves in high-frequency regions (unless stated as audible), which are detected and measured by a sensor that converts an equivalent stress magnitude into a corresponding electrical signal [15]. A visual illustration is shown in Figure 2, while an illustration of the acoustic spectrum can be seen in Figure 3.

Figure 2 shows a source event, indicated by S, undergoing some form of deformation, which culminates in the release of energy in the form of stress waves. These are shown as arrows pointing away from the source and ultimately acquired by the biosensor, which records the entire acoustic device and is saved within the supporting electronic module.

Figure 3 shows the various groups of AE regions across the frequency spectrum, consisting of low frequencies, audible acoustics centred on human hearing, and higher frequencies, which are the regions where joint pathologies have been seen to be centred.

The output electrical signal—which is typically measured in voltage V(t) and gives an indication and magnitude of the AE signal and event itself—is a function of a convolutional process that has three sub-dependencies, namely: (1) the source function S(t), which is related to the source AE event itself; (2) the wave propagation medium G(t); and (3) the acquisition characteristics of the recording instrument, which is termed as the instrument response function, mathematically expressed as Equation (1):

(1) V(t)=S(t)∗G(t)∗R(t)

**Figure 4:**
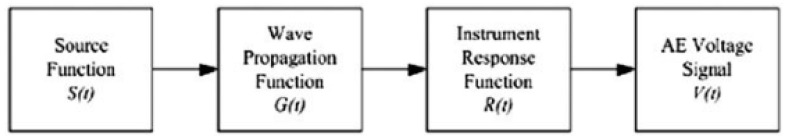
Signal shaping chain [18].

For scenarios where the tissue characteristics do not vary considerably, the stress waves from the source event and the instrument response function from the acquisition electronics remain constant. It can be presumed that a pseudo-linear relationship exists between the source acoustic event and the concurrent magnitude of the output voltage signal. Diagrammatically, this can be represented as seen in Figure 4.

## 3. Tribological Surface Mechanics

Tribology is the science of surfaces that rub and are in contact with each other, which serves as an equivalent scale-down and theoretical basis towards the study of biotribological and orthopaedic conditions that can occur in the joints of a human being, assuming a set of surfaces that have contact and an apparent interaction that can span nano/macro-scale contact depending on the level and scale of the interaction [19].

Di Puccio and Mattei [19] described the contact forces, assuming a macro-scale interaction, as ideal bodies that are elastic under loading conditions and that undergo a form of elastic deformation that can be physically governed using the principle of Young’s modulus. For a scenario involving contact with two surfaces assumed to be perfect spheres, an analytical solution can be obtained using Hertz’s theory of contact, which considers the contact pressure and area for two spheres in contact [20]. Although Hertz’s proposition is for nonconformal bodies of two spheres, its principles can be extrapolated forward for a scenario involving biological joint contact [20].

The friction during the process is a resistance source between the two bodies in contact, which, as per Di Puccio and Mattei [19], can either be rolling or sliding. These can coincide where the friction force is likely due to adhesions and surface deformations at contact junctions [19]. In order to minimise the effect of friction on contacting bodies in biological joints, a lubrication source (synovial fluid) usually is present to promote a safe and sustainable range of motion [19]. This lubrication source is optimally placed between contact bodies to minimise the asperities and frictional force between them [19].

In this paper, the following five joint pathological conditions are examined and investigated with the aid of AE sensing.

**Figure 5:**
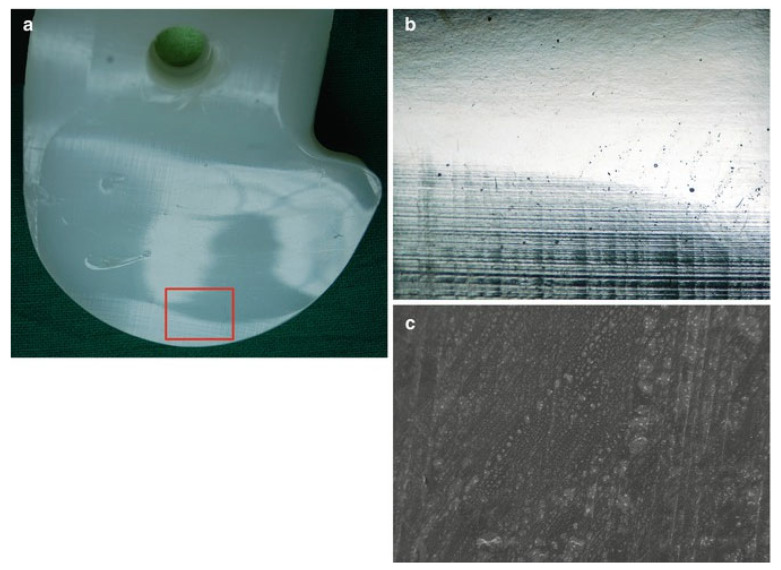
Tibial polyethylene with successive higher resolutions. In (**a**), the highlighted rectangle is zoomed in and can be seen in (**b**), showing a series of horizontal wear marks at the bottom, while (**c**) is a scanning electron microscopic image that further shows the machining marks on the surface of the material [22].

**Figure 6:**
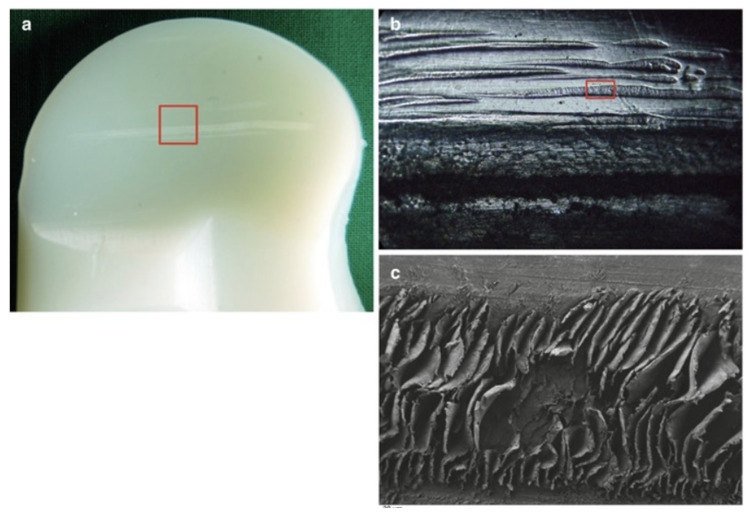
(**a**) Image of a polyethylene material with a red rectangle, which is zoomed into for (**b**) with a further magnification of the scratched area, while (**c**) represents a scanning electron microscopic image in high resolution, further providing a visual illustration of the concept [22].

(1) Abrasive wear involves the concept of abrasion and refers to the scratching and rubbing motion between a set of contact bodies.

(2) Adhesive wear is generated under plastic contact and ultimately leads to a fracture due to the adhesion between the two surfaces. It thus represents a kind of wear that occurs when the atomic force between a set of materials under a loading condition supersedes the apparent properties of the material surface [21]. It is common for particles to form a by-product of the wear between two surfaces, typically called ‘wear particles’ [21].

(3) Burnishing wear involves the systematic plastic deformation of a surface by another due to prolonged and continuous sliding contact, during which surface smoothening creates a glare-like reflection of the burnished surface. The mechanical principle is based on the induced localised contact stress exceeding the overall yield strength of the material. A series of images illustrating this concept for a candidate tibial polyethylene material can be seen in Figure 5.

(4) Burnishing to scratching is a wear type that represents an intermediary and transition between two types of wear and thus carries a superimposed characteristic of both the burnishing and the scratching wear.

(5) Scratching is a wear type caused by advanced abrasion and involves ploughing a material’s surface, which results in the alteration of the material surface with a series of lines along the direction of the stress [22]. Images of this kind of wear can be seen in Figure 6 below.

## 4. Data Acquisition

**Figure 7:**
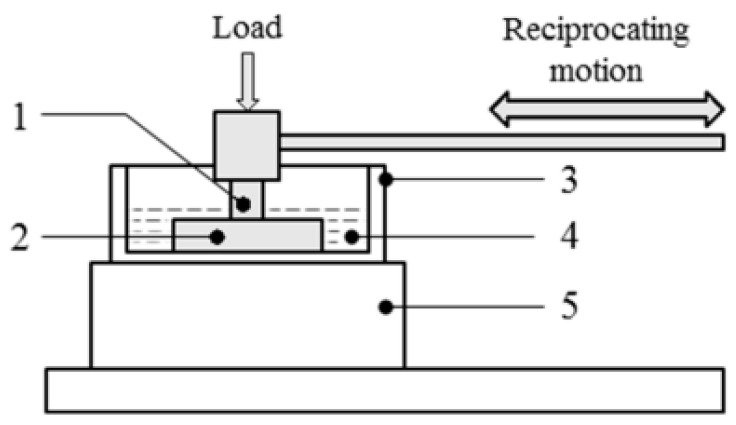
Schematic view of the TE77 tribometer setup: (1) PEEK upper specimen and AE sensor, (2) steel lower specimen, (3) lubricant bath, (4) lubricant (Ringer’s solution), (5) heater block [15].

The data collection sequence employed in this paper follows the sequence described by Olorunlambe et al. [15], where AE signals were acquired in a setup comprising a tribometer and an AE signal acquisition platform, which is designed to simulate the various wear conditions described in the prior section. An image of the experimental layout can be seen in Figure 7 below.

The data collection description was chunked into two significant parts reflecting the kinds of fault simulations, as follows.

- Abrasion and adhesive wear: sliding tests were done with the TE77 high-frequency reciprocating machine with a cylinder-on-plate configuration. The tests were conducted with a polyetheretherketone (PEEK) rod as the reciprocating specimen, while a steel plate served as the fixed lower specimen [15]. The candidate materials were explicitly selected to replicate a metal form on polymer joint articulation. All plates were cleansed in ethanol prior to experiments and, in particular, were roughened with a belt sander attached with P40 grade sandpaper to mimic an abrasive wear condition. Quarter-strength Ringer’s solution was used as the lubricant for this set of tests [15].

The initial load tests were calculated using the Hertzian theory for contact mechanics, assuming a ball-and-socket Charité lumbar spinal implant with a 10 mm ball radius and 0.35 mm radial clearance, with loading and displacement conditions as described by BSISO 18192-1 for wear conditions of total intervertebral spinal disc prostheses. Further test parameters included a load of 150 N, frequency of 2 Hz, a stroke of 12.5 mm, AE acquisition threshold of 40 dB, preamplifier gain of 60 dB, and bandpass filter of 100–600 kHz.

- Burnishing, burnishing to scratching and scratching: the same experimental conditions as above were adopted for these experiments. The tests were conducted with an ultra-high-molecular-weight polyethylene (UHMWPE) disc as the reciprocating sample, with medical grade cobalt chromium molybdenum alloy (CoCrMo) as the fixed specimen. The dimensions of the UHMWPE disc were a diameter of 10 mm with a 3 mm thickness, which was subsequently machined to a fine surface finish of 0.65 ± 0.17 μm. These test conditions were replicas of the linear motion of a set of hinged knees as established using appendix A1 of ASTM F732-17 loading conditions, while the recommended contact pressure of 3.54 MPa was doubled in an attempt to simulate variants of severe wear damage. The burnishing wear was simulated using the UHMWPE continuously sliding on the CoCrMo plate, while the scratching wear was simulated with 45 mg of 80-grit-size silicon carbide with grinding grit added to the contact surface between the UHMWPE disc and the CoCrMo plate before testing.

**Figure 8:**
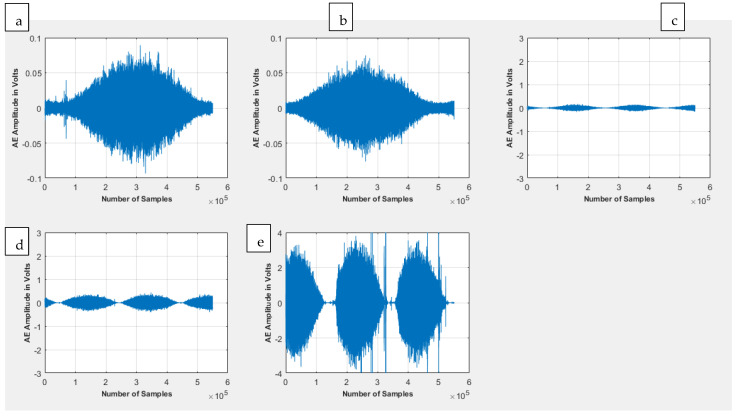
A visual image of the various kinds of signals considered as part of this work: (**a**) abrasive wear, (**b**) adhesive wear, (**c**) burnishing wear, (**d**) burnishing to scratching transitional signal, (**e**) scratching wear.

A time-domain visualisation of the various kinds of signals can be seen in Figure 8 below.

From Figure 8, a qualitative view of the various wear signatures produced various time-series patterns. From this, it can be seen that a snapshot view of the abrasive wear produces a signature that resembles an enveloped pattern with a unique rise time followed by a peak and then a transient decay. The same appears to be the case for the adhesive wear owing to the similarity of the two sets of wear classes, with the critical differences between them being the reduced overall amplitude of the adhesive wear signal itself, with a reduced level of “roughness” of the resulting amplitude waveform when compared to that of the abrasive wear. The burnishing wear exhibits many cyclical envelopes per time frame compared with the abrasive and adhesive wear, indicative of much more dynamic activity in a much shorter time frame. The subsequent signal represents an intermediary and transitional burnishing to the scratching wear signal and exhibits a nearly identical pattern to the prior burnishing signal, albeit with a slightly higher amplitude. The final scratching wear signal from the transition is characterised by the same multiple dynamic events within the same time frame relative to the first two conditions, albeit this time with a much more intense amplitude, which is symbolic of the extreme activity that occurs during this wear scenario.

### AE Signal Conditioning and Post-Processing

AE signals were acquired using an acquisition and recording system (supplied by the Mistras Group, Cambridge, UK) comprising a nano-30 AE sensor, a 2/4/6 preamplifier with a gain of 60 dB, and the AEWin PCI2 software [23]. The sensor was fixed to the upper specimen holder (Figure 7, item (1)), connected to the preamplifier and then to a computer with the software installed for signal conditioning and acquisition. Signals were acquired at a sampling frequency of 2 MHz throughout the tests.

## 5. Methods

### 5.1. Data Preprocessing

In order to replicate the online process that the final model is intended for, the data were preprocessed and windowed in a specific format, where—primarily due to the high sampling rate of the AE signal—1,000,000 samples were used for the model build process. These data were windowed using a disjointed window scheme of 10 equal slices, which ultimately amounted to 100,000 samples per window.

### 5.2. Feature Extraction

The feature group utilised as a part of this study represented an expanded group of features seen in previous studies to characterise and contribute towards the effective modelling and pattern recognition of stochastic nonlinear signals, in addition to physiological signals [24,25,26,27]. The group represented a concatenation spanning statistical, frequency, nonlinear and fractal-based features. Table 1 shows the comprehensive list of features, their characteristics and associated parameters, where relevant.

### 5.3. Deep Wavelet Scattering (DWS)

DWS is a multiresolution approach that combines properties from wavelet decomposition and the deep learning-based convolutional neural network (CNN). The concept of the DWS allows for an unsupervised feature extraction where the features are continuous and robust to any translations. The significant difference here involves using preset wavelets and scaling filters instead of a data-driven iterative learning process [28,29,30,31,32].

**Figure 9:**
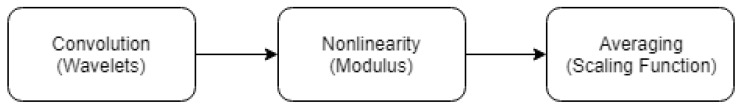
The key associated steps of deep wavelet scattering [31].

DWS is a result of seminal contributions from several researchers, including Mallat et al., who provided a mathematical formalism of the CNN and how its properties contribute towards the overall functionality of DWS, which include multiscale contractions, the linearisation of hierarchical symmetries, and sparse representations [28,29,30,31,32,33]. One of the critical differences and apparent strengths of DWS relative to the standard CNN is based on being able to work with a smaller sample set due to not needing to iteratively learn its values for its filters [28,29,30,31,32,33]. A flow diagram illustrating the various substages of the DWS can be seen in Figure 9.

In terms of mathematical formalisms, given a signal f(t) being analysed with Ø being a low-pass filter and a wavelet function of Ψ for filtering purposes, whose range spans the range of frequencies of the signal, the wavelet family indices possess an octave frequency resolution $Q_{k}$ and are denoted as $\wedge_{k}$, as well as the multiscale high-pass filter banks ${\left\{\Psi_{\mathrm{jk}}\right\}}_{jk\in \wedge_{k}}$, which are constructed via dilation of the wavelet Ψ. Furthermore, ØJ(t) is a low-pass filter that provides a localised translation invariance of f at a defined scale T [28,29,30,31,32].

As mentioned, the DWS was implemented as a wavelet scattering network with the use of a CNN, which sequentially performs convolutions first through classical wavelets, then a nonlinear modulus, and is tailed off with an averaging scaling function, as indicated by the flow diagram in Figure 9 [28,29,30,31,32]. The process of convolutions indicated by $S_{0}f\left(t\right)=f*{Ø}_{J}\left(t\right)$, where $S_{0}$ is the zero-order scattering coefficients, produces locally invariant translation features of f, which, although at first result in a temporary loss of high-frequency information, can be dually recouped via the wavelet modulus process $\left|W_{1}\right|$, as expressed in Equation (2) [28,29,30,31,32]:

(2) $$\left|W_{1}\right|f={\left\{S_{0}f\left(t\right),\left|f*\Psi_{j1}\left(t\right)\right|\right\}}_{j1\in \wedge_{1}}$$

Hierarchically, the first set of scattering coefficients can be obtained via an averaging process of the wavelet modulus coefficient ${Ø}_{J}\left(t\right)$, as seen in Equation (3):

(3) $$S_{1}f\left(t\right)={\left\{\left|f*\Psi_{j1}\left(t\right)\right|\;*{Ø}_{J}\left(t\right)\right\}}_{j1\in \wedge_{1}}$$

A certain amount of information is lost from the averaging process, which once again can be recovered via a mathematical transformation ($S_{1}f\left(t\right)$, which can be seen to be the low-frequency constituent of $\left|f*\Psi_{j1}\right|$) via the application of the wavelet modulus as part of the information recovery process, as shown as $\left|W_{2}\right|\left|f*\Psi_{j1}\right|={\left\{S_{1}f\left(t\right),|\left|f*\Psi_{j1}|*\Psi_{j2}\left(t\right)\right|\right\}}_{j2\in \wedge_{2}}$ [28,29,30,31,32].

Whilst the second-order coefficient can be defined as $S_{2}f\left(t\right)={\left\{\left|\left|f*\Psi_{j1}\right|*\Psi_{j2}\right|*{Ø}_{J}\left(t\right)\right\}}_{j1\in \wedge_{1}}\;i=1,2,$ a continuous iteration via the defined process yields the following wavelet convolutions as Equation (4):

(4) $$U_{m}f\left(t\right)={\left\{\left|\left|f*\Psi_{j1}\right|*\ldots .|*\Psi_{\mathrm{jm}}\right|\right\}}_{j1\in \wedge_{1}},i=1,2,\ldots m.$$

where $U_{m}$ is an *m*th-order modulus.

How to obtain the *m*th-order scattering coefficient, $U_{m}f\left(t\right)$ with ${Ø}_{J}$ can be seen as in Equation (5):

(5) $$S_{m}f\left(t\right)={\left\{\left|\left|f*\Psi_{j1}\right|*\ldots .|*\Psi_{\mathrm{jm}}\right|*{Ø}_{J}\left(t\right)\right\}}_{j1\in \wedge_{1}},i=1,2,\ldots m$$

**Figure 10:**
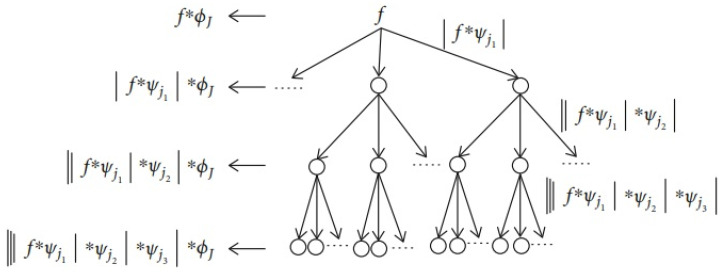
A tree-based visualisation of the scattering decomposition network [33].

The defined approach is used to obtain a final scatter matrix $Sf\left(t\right)={\left\{S_{m}f\left(t\right)\right\}}_{0\leq m\leq l}$, which concatenates the scattering coefficients from all orders to characterise an input signal, where l represents the maximum decomposition level [28,29,30,31,32]. A tree-based visualisation of the scattering decomposition network can be seen in Figure 10.

As described, the DWS retains the simultaneous characteristics of both the CNN and the wavelet by exhibiting translation invariance while also being stable to local deformations [28,29,30,31,32]. As far as the critical differences between them, the filter parameters are preset in DWS and thus negate the need for subsequent iterative learning of the optimal parameters, in addition to the final output not being solely from the output layer but rather a combination from multiple preceding layers [28,29,30,31,32].

As the energy of the resulting scatter coefficients has been seen to attenuate with an increasing number of network layers, with the bulk of the energy hosted in the first layers, as a result of this, a two-order scattering network configuration would be adopted in this work, as adapted from related studies [28,29,30,31,32]. The other supporting configuration parameters used towards running the DWS include the use of the Gabor wavelet as the principal mother wavelet for the decomposition actions, with an invariance scale of 1 s, alongside a series of filter banks spanning eight wavelets per octave in the first filter bank followed by one wavelet per octave in the second filter bank.

**Figure 11:**
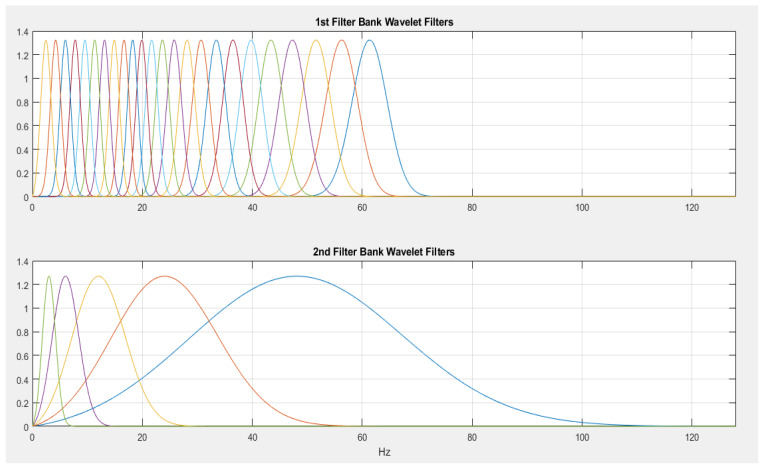
The first filter bank with eight wavelets per octave.

**Figure 12:**
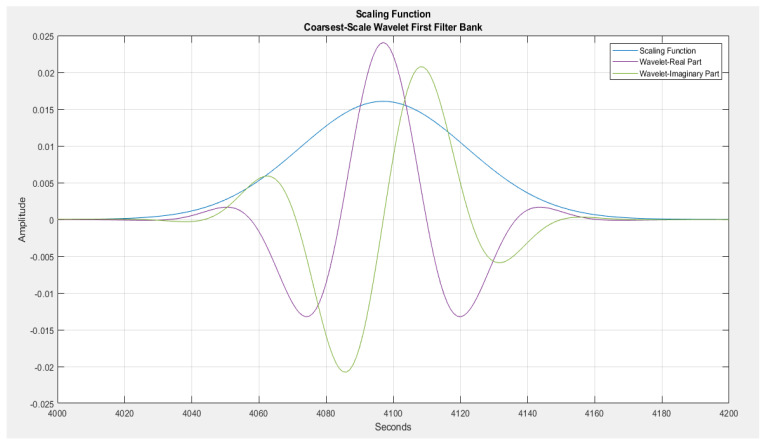
The low pass filter with 1 s invariance scale.

Images of the various filter banks from the two network layers alongside a low-pass filter with a 1 s scale invariance can be seen in Figure 11 and Figure 12.

### 5.4. Prediction Machines

Decision Tree (DT): this model is nonparametric and works with Boolean logic towards distinguishing and sorting incoming data into their various classes in a hierarchical fashion reminiscent of a tree [34]. This model is regarded as a grey-box-type modelling approach due to carrying a specific level of transparency and, therein, model decision explainability [34].

Discriminant Analysis (DA): this is a computationally efficient model underpinned by statistical reasoning. The model works with a projection of the candidate feature vector into a lower dimensional subspace and is followed by the placement of decision boundaries to separate the various classes [24]. The linear version of the model was utilised in this paper, i.e., linear discriminant analysis (LDA).

Kernel Naïve Bayes (KNB): the naïve Bayes classifier works with probabilistic reasoning, which stems from Bayes’s theorem alongside different independence assumptions. Unlike its counterparts in the literature, it has been seen to work well with relatively fewer data, mainly due to its empirical formalism, as opposed to an iterative one [35]. The implemented configuration of the naïve Bayes classifier utilises a kernel, a nonparametric estimation method used to identify random variable density functions [35].

Support Vector Machine: these models are of an iterative configuration finding a solution to an optimisation problem of finding the best location of the separation boundary between data classes whilst utilising a portion of the data, referred to as the support vectors [36]. SVM typically involves the projection of the data into a higher dimensional subspace whilst working with the notion that data classes have a greater degree of separation in a higher dimensional subspace, followed by the placement of the various decision boundaries [36]. The data are subsequently projected back down into a lower dimensional space whilst continuously preserving the overall structure of the data and the locations of the class boundaries in a process referred to as the ‘kernel trick’ [36]. Six different configurations of the SVM were used in this paper, namely, the linear support vector machine (LSVM), quadratic support vector machine (QSVM), cubic support vector machine (CSVM), fine Gaussian support vector machine (FGSVM), medium Gaussian support vector machine (MGSVM), and coarse Gaussian support vector machine (CGSVM).

K-Nearest Neighbour (KNN): this represents a category of nonparametric-based classification models with a majority vote on the nearest neighbour. Here, K is chosen as one, and the model is based on the association of a sample to a data class using the sole nearest neighbour and is a computationally efficient configuration of the model [37]. The Euclidean distance criteria were selected as the distance index for this work, and three different variants of the model were used, namely, fine KNN (F-KNN), medium KNN (M-KNN) and coarse KNN (C-KNN).

The design and validation of all listed prediction machines were performed with the MATLAB Classification Learner app, which automatically tuned and optimised all models to achieve their optimal parameters. All models were validated using a K-fold cross-validation approach, with K chosen as 10 and a data split format of 80:20, with 80% used towards the model’s training, while the remaining 20% was utilised towards the validation of the designed models.

## 6. Results and Discussion

**Figure 13:**
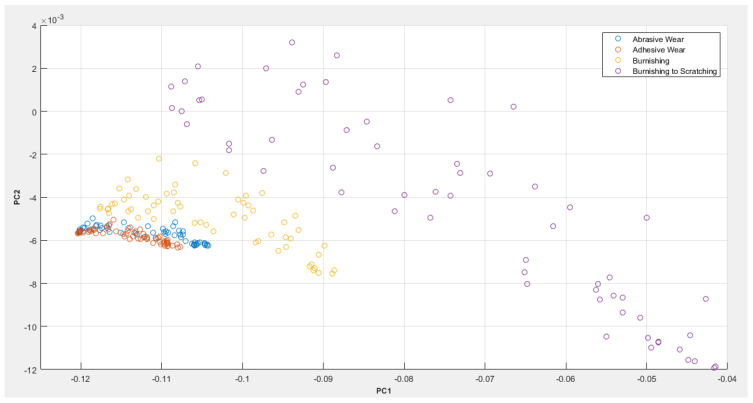
PCA plot of the DWS of four pathology cases: abrasive wear, adhesive wear, burnishing and burnishing to scratching.

Figure 13 is a principal component analysis (PCA) plot of the DWS of the four pathology cases considered in this study.

From the PCA plot, it can be seen that although there exists a cluster separation between the abrasive and adhesive wear, they appear to be close to each other, primarily due to the similarities of their AE signal waveforms, as shown in Figure 8. The burnishing condition seems distinctly separate from the abrasive and adhesive conditions on the PCA plot, while the burnishing-to-scratching case appears separate from the rest of the samples’ clusters. There also exists a high degree of separability within the samples of the burnishing to the scratching cluster itself.

The results for the prediction exercises can be seen in Table 2 across the range of models investigated. In the case of the raw/handcrafted features, the mean performance across all 12 models produced an accuracy figure of 90 ± 7.16%, which produced a range of accuracies. The LDA, alongside distinct configurations of the SVM, resulted in the best model prediction accuracies. The DWS produced a mean accuracy across all classifiers of 97 ± 3.77%, up from the raw/handcrafted features figures and provides evidence to suggest multiscale-based characterisation of the AE signals appears to provide the best signal performance under the conditions that have been investigated. These results echo the findings from previous AE-based studies, which have utilised decomposition methods towards analysing acquired AE signals. The DWS also offers the flexibility of not requiring expert-based knowledge due to its unsupervised feature learning scheme. Although this comes at a higher computational cost, it allows for an ensemble of signal decomposition and feature characterisation in a fused fashion. Subsequent studies would investigate the effect of low dimensional embedding methods on helping speed up the modelling process involved with the DWS.

**Figure 14:**
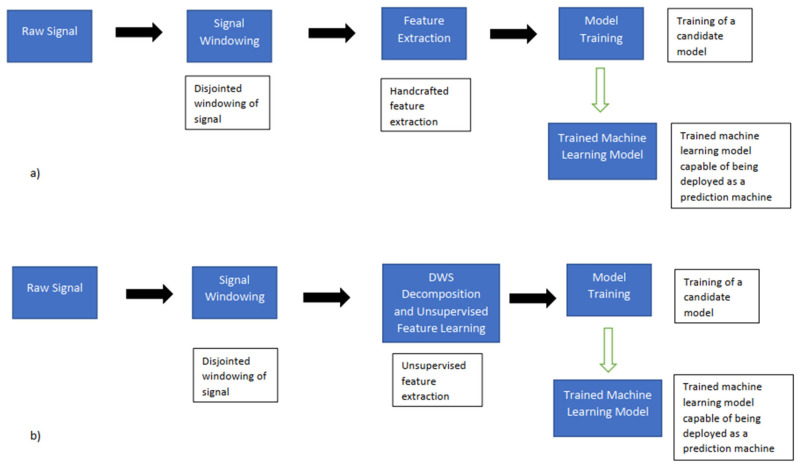
(**a**) Modelling pipeline using raw/handcrafted features, (**b**) pipeline using DWS/unsupervised feature learning.

Figure 14 is a flow diagram comprising the various phases associated with the training and design of a candidate prediction machine poised towards predicting various joint pathologies. The critical difference between the pair of modelling flows is that for the raw/handcrafted features, prior expert knowledge is necessary to assemble and extract the relevant group of signal features, which can be used towards characterising the signal [38]. Conversely, in the case of the DWS, an unsupervised multiscale feature extraction scheme is adopted, for which only parameters are to be defined for the process before the signal is characterised in an unsupervised fashion.

Table 3 shows and contrasts the various properties of handcrafted feature extraction and DWS unsupervised feature extraction.

### 6.1. Feature Ranking Results

In order to observe the critical features from the group of handcrafted features, which are the key drivers towards the modelling and characterisation of the resulting AE signals, the Relief feature ranking algorithm was used for the ranking of the importance of the various features, as seen in Table 4 [39,40]. It should be noted that this was not possible for the DWS due to the lack of feature interpretability, and therein represents a strength of handcrafted features over unsupervised feature learners in general.

From the results in Table 4, it can be seen that three of five of the top-ranked features are frequency-based features in the form of the PF, MF and SSC, which show the importance of frequency-based characterisation and information for the modelling of these kinds of AE signals. The remaining two features are from the nonlinear literature in the form of the two fractal-based features that can be seen in Table 4, which echoes their strength and importance with a view towards dealing with nonstationary time-varying signals.

### 6.2. Extension towards a Real-Time Diagnosis System

**Figure 15:**
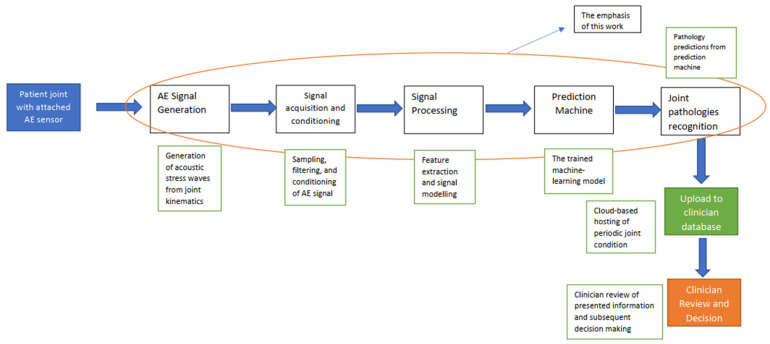
A proposed flow of the remote clinical decision-support module for the monitoring of joint pathologies, from AE signal acquisition towards final joint-wear prediction.

The envisaged pathway towards a mobile joint health monitoring platform that can be utilised for the continuous monitoring of patient joint pathologies can be seen in Figure 15 [41,42]. The method commences with an ergonomically placed AE sensor on the specific joint of interest on the patient’s body, recording the passive acoustic stress waves from the kinematic motions of the joint during daily activities. Upon acquisition of these signals, the usual digitisation process is expected to take place as part of the signal acquisition and conditioning process. The signal characterisation follows processing via either the handcrafted features or unsupervised, depending on the embedded computational hardware utilised as a part of the overall system. A feature vector would then be fed towards a trained machine learning model—which in this case is serving as an online prediction machine, and based on trained examples—that is expected to proceed to make a prediction based on the joint pathology and potentially the extent of the joint wear (subject for future work) based on input data [43]. This prediction is ultimately passed and ported to the clinicians’ cloud-based system, where it remotely receives an update on the overall health of the patient’s joint. Using this, alongside reviewing the patient’s clinical information, the clinicians can decide what course of clinical action to take subsequently [44]. Thus, from a broad view, the proposed AE-driven joint pathology system can be referred to as a remote clinical decision-support platform for monitoring joint pathologies.

## 7. Conclusions and Future Work

The condition of joint wear is shaping to become a broader-scale medical issue, amplified by the projections of the ageing population in the years to come. On top of this, athletes who generally play impact sports have been seen to be susceptible to various joint pathologies. The current means of assessing and diagnosing joint wear and pathologies currently span X-ray, CT and MRI, all of which have technical shortcomings and are primarily unaffordable to developing nations within their medical settings.

AE presents an alternative to the modalities above in that it is a mobile sensing method that makes passive recordings during the natural joint movement of the patient while presenting no risk of radiation absorption to the patient. The primary challenge associated with using the modality is based on the considerable amount of signal processing modelling required to model and decode the AE events from the resulting source events. In this paper, we have contrasted two independent signal processing methods alongside 12 machine learning models for identifying and differentiating five kinds of simulated joint pathologies, where a visual and qualitative view of the AE from the five joint pathologies showed distinct differences in the various signals.

The two contrasting signal processing methods employed for modelling the mixed AE signals included using an expanded set of handcrafted features comprising statistical and nonlinear features, all the way towards complexity and fractal features. At the same time, the second approach utilised a deep learning-based method in the form of the DWS, which allowed for an unsupervised feature learning mechanism. The results for both candidate methods across the various machine learning models showed that the DWS provided the best prediction and differentiation prowess across the various signal processing methods considered. The main downside of this method is the relatively tricky computational time required for its processing, mainly due to the multiscale nature of the algorithm itself.

The proposed signal processing model fits into a broader system for remote condition monitoring of joint pathologies, as shown in Figure 15, which is poised to be a mobile/remote clinical decision-support platform to aid clinicians towards taking proactive decisions regarding the care and treatment of patients with joint pathologies. Potential future work in this area would involve the expansion of the current model to be able to detect the wear extent of a specific identified joint pathology in order to support the clinician towards prioritising care and inferring the stage and extent of the joint pathology and which would allow for the use of regressions [45,46,47].

## Figures and Tables

**Table 1:** List of features, their characteristics and associated parameters (where necessary).

Feature	Characteristic	Parameters (Where Necessary)	References
- Mean Absolute Value (MAV)	- provides a quantification of the mean amplitude and intensity of a signal	- n/a	[24,25,26,27]
- Waveform Length (WL)	- this feature quantifies the cumulative length of a waveform over a defined segment, which can provide insight into characteristics such as the amplitude, frequency, and duration	- threshold = 0.000001	
- Zero Crossing (ZC)	- represents a robust feature which indicates how many times the waveform crosses a predefined threshold	- threshold = 0.000001	
- Slope Sign Change (SSC)	- working concerning a threshold, this feature indicates the changes in the polarity of the slope values across a set of three consecutive samples	- threshold = 0.000001	
- Root Mean Squared (RMS)	- gives a quantified reflection of the amount of power	- n/a	
- 4th Order Autoregressive Coefficient (4th AR)	- is a time-series analysis-based model which linearly combines and regresses estimations of previous samples and has proved to be effective in the differentiation of time-varying signals that have a varying mean-amplitude	- n/a	
- Sample Entropy (SampEN)	- is a feature which indicates the regularity and complexity present within a signal	- m = 2, r = 0.2	
- Max Cepstrum Component (Ceps)	- this feature deconvolves a signal into a spectrum, following which the maximum value is obtained	- n/a	
- Maximum Fractal Length (MFL)	- is a stochastic complexity-based feature which calculates the level of power within a signal	- n/a	
- Higuchi Fractal Dimension (HFD)	- is a complex feature which is computationally used towards calculating the fractal dimension of a signal	- Kmax = 10	
- Detrended Fluctuation Analysis (DFA)	- is a complexity-based feature towards computing the degree, level, self-similarity and affinity of a signal	- 30:10:300	
- Median Frequency (MF)	- is a frequency domain-based feature which extracts the medium frequency value across a candidate frequency spectrum	- n/a	
- Peak Frequency (PF)	- is a frequency domain-based feature which extracts the peak/maximum frequency value across a candidate frequency spectrum	- n/a	
- Number of Peaks (NP)	- this feature provides a cumulative sum of the number of peaks detected based on a peak detection heuristic algorithm	- threshold = 0.000001	
- Simple Squared Integral (SSI)	- provides an alternate perspective towards capturing the power existing within a signal	- n/a	
- Variance (VAR)	- provides an indication of the sample variation and overall spread of the values within a candidate set of values	- n/a	

**Table 2:** Results comparison table.

Model	Raw Features (%)	DWS (%)
DT	82	98
LDA	100	98
KNB	78	99
LSVM	94	99
QSVM	94	99
CSVM	96	100
FGSVM	90	87
MGSVM	96	98
CGSVM	92	91
FKNN	96	100
MKNN	80	98
CKNN	82	98
Mean across all models	90 ± 7.16	97 ± 3.77

**Table 3:** A comparison table contrasting the characteristics and performance of the handcrafted method with the DWS.

Handcrafted Features	DWS
- Relies on expert knowledge to source out the feature set	- Unsupervised feature extraction, thus does not rely on expert knowledge
- Less computationally intense/dependent on feature group	- Computationally intense
- Feature groups can be varied	- Distinct multiscale features
- Suboptimal performance with small samples	- Works well with a small set of data samples
- Mostly algorithmically structured	- Based on a fusion of the properties of both the CNN and wavelet transform

**Table 4:** Handcrafted features ranked in order of importance towards the modelling and characterisation of the resulting AE signals.

Feature Ranking Results
(1) Peak Frequency (2) Median Frequency (3) Detrended Fluctuation Analysis (4) Higuchi Fractal Dimension (5) Slope Sign Change

## Data Availability

The data are available from the authors upon reasonable request.
